# Phenotypic and Functional Dysregulated Blood NK Cells in Colorectal Cancer Patients Can Be Activated by Cetuximab Plus IL-2 or IL-15

**DOI:** 10.3389/fimmu.2016.00413

**Published:** 2016-10-10

**Authors:** Yamila Sol Rocca, María Paula Roberti, Estefanía Paula Juliá, María Betina Pampena, Luisina Bruno, Sergio Rivero, Eduardo Huertas, Fernando Sánchez Loria, Alejandro Pairola, Anne Caignard, José Mordoh, Estrella Mariel Levy

**Affiliations:** ^1^Fundación Instituto Leloir-IIBBA, Ciudad Autónoma de Buenos Aires, Buenos Aires, Argentina; ^2^Centro de Investigaciones Oncológicas CIO-FUCA, Ciudad Autónoma de Buenos Aires, Buenos Aires, Argentina; ^3^Instituto Alexander Fleming, Ciudad Autónoma de Buenos Aires, Buenos Aires, Argentina; ^4^UMRS-1160, Institut National de la Santé et de la Recherche Médicale (INSERM), Paris, France; ^5^U1160, Université Paris Diderot, Sorbonne Paris Cité, Paris, France

**Keywords:** natural killer cells, colorectal cancer, NKp46, NKp30, cetuximab, IL-2, IL-15

## Abstract

The clinical outcome of colorectal cancer (CRC) is associated with the immune response; thus, these tumors could be responsive to different immune therapy approaches. Natural killer (NK) cells are key antitumor primary effectors that can eliminate CRC cells without prior immunization. We previously determined that NK cells from the local tumor environment of CRC tumors display a profoundly altered phenotype compared with circulating NK cells from healthy donors (HD). In this study, we evaluated peripheral blood NK cells from untreated patients and their possible role in metastasis progression. We observed profound deregulation in receptor expression even in early stages of disease compared with HD. CRC-NK cells displayed underexpression of CD16, NKG2D, DNAM-1, CD161, NKp46, and NKp30 activating receptors, while inhibitory receptors CD85j and NKG2A were overexpressed. This inhibited phenotype affected cytotoxic functionality against CRC cells and interferon-γ production. We also determined that NKp30 and NKp46 are the key receptors involved in detriment of CRC-NK cells’ antitumor activity. Moreover, NKp46 expression correlated with relapse-free survival of CRC patients with a maximum follow-up of 71 months. CRC-NK cells also exhibited altered antibody-dependent cellular cytotoxicity function responding poorly to cetuximab. IL-2 and IL-15 in combination with cetuximab stimulated NK cell, improving cytotoxicity. These results show potential strategies to enhance CRC-NK cell activity.

## Introduction

Colorectal cancer (CRC) is the second leading cause of cancer mortality in the U.S., with approximately 143,000 new cases and 51,000 deaths expected by year (1). From the diagnosis of stage I and II cases, surgical resection is considered the mainstay treatment, with a 5-year survival rate of 80% (2). The standard management of advanced CRC has historically focused on multi-agent chemotherapy. More recently, development of monoclonal antibodies (mAbs) that target VEGF (bevacizumab) and EGFR (cetuximab) led to their application in metastatic patients (stage IV) (3–5). Still, 70% of relapsed patients have a life expectancy of less than 2 years, as they do not respond to conventional therapy (6). Unlike conventional chemotherapies and targeted anticancer agents, which act directly on malignant cells or proximal stromal cells, immunotherapies have an indirect mechanism of action (7). It is clear that besides melanoma and renal cell carcinoma, traditionally considered immunologic targets, other solid tumors can also be immunologically responsive. Thus, the immune response in CRC is associated with clinical outcome (8), and it is known that CRC cells remain susceptible to T-cell-mediated killing despite prior exposure to chemotherapy (9). Innate immunity is involved in tumor immunosurveillance, and cytotoxic natural killer (NK) cells play an important role in the immune response to cancer by recognizing and killing a wide variety of tumor cells with reduced or absent MHC-class I expression or the expression of stress-induced molecules (10).

Natural killer cells exert direct cytotoxicity through perforin and granzyme exocytosis and by engagement of death receptors. NK cells also regulate the immune response by cytokine production such as interferon-γ (IFN-γ) or tumor-necrosis factor-α (TNF-α) (11–13). NK cell activation is controlled by a dynamic balance between the positive and negative signals trigged by both activating and inhibitory receptors, correspondingly (10–12, 14, 15). In the activating group, NKp30, NKp44 and NKp46 natural cytotoxicity receptors (NCR), NKG2D, DNAM-1, CD161, and CD16 receptors favor cytotoxicity and/or the production of cytokines. In the second group, NKG2A, CD85j, and CD158b receptors provide suppression signals by inhibition of stimulatory pathway (13, 16, 17). Recent evidence suggests that NK cells may also shape the adaptive immune response toward a T helper 1 (Th1) profile, which is thought to be a key component in antitumor responses (18, 19). However, NK cell efficacy against human solid tumors remains questionable, possibly due to the tumor microenvironment which suppresses the availability of fully competent NK cells at the tumor site (20). We previously determined that NK cells from CRC patients displayed multiple alterations in their phenotype with a drastic reduction in the expression of NK cell-activating receptors (21). Similarly, peripheral blood (PB) analysis revealed NK cells dysfunction with reduced expression of activating receptors (22) and recently Hou et al. (23) determined that the intracellular IFN-γ production and degranulation activity of NK cells in patients with CRC were significantly lower than the one in healthy adults.

In the present work, we evaluated PB NK cells from untreated CRC patients, and found a significant dysregulation in patients from all clinical stages, including those with limited disease that affects functionality against CRC cells. More precisely, NKp30 and NKp46 were the main receptors involved in CRC-NK cell inhibition of antitumor activity. Moreover, the downregulation of NKp46 receptor expression correlated with lower relapse-free survival (RFS) of CRC patients with a maximum follow-up of 71 months. CRC-NK cells also exhibited altered antibody-dependent cellular cytotoxicity (ADCC) function responding poorly to cetuximab, which in addition to inhibiting the proliferation of K-RAS wild-type tumor cells (24), mediates ADCC performed by NK cells through CD16 Fcγ receptor, which has been strongly associated to cetuximab response in CRC (25). Finally, in order to reactivate CRC-NK cells, we assayed the combination of IL-2 and IL-15 cytokines with cetuximab resulting in an *in vitro* improved antitumor activity.

## Materials and Methods

### Patient Samples

The present study was approved by the Institutional Ethics Committee of the Instituto Alexander Fleming (IAF), and all patients enrolled provided written-informed consent. Samples were obtained from 52 patients (AJCC stages I–IV) without any other concomitant colorectal disease who underwent surgical resection of CRC at the Surgery Service of the IAF (Table 1). Inclusion criteria: written-informed consent, age ≥18 years old, and available blood sample collected at the moment of surgery. Exclusion criteria: exposure to chemotherapy and/or lack of written consent. As blood samples were limited in some cases, some determinations could not be performed for all patients. Nine of them where only assayed for TGF-β measurement, and functional assays were performed in a reduced number of samples. As controls, PB samples were obtained from healthy donors (HD) at the Hemotherapy Service of the IAF.

**Table 1:** **Clinical and histological characteristics of CRC patients**.

Patients characteristics		
Gender	Male	26
	Female	26
Age	<50	10
	≥50	42
Localization	Right/trasversum colon	12
	Left colon	28
	Rectum	12
Stage	I	8
	II	23
	III	13
	IV	8
Histological grade	Well differentiated	43
	Poorly differentiated	9

### Collection of Samples and NK Cell Isolation

Peripheral blood samples from CRC patients (3–15 ml each) and HD were obtained in heparinized collection tubes. PB mononuclear cells (PBMC) were isolated by Ficoll-Paque PLUS (GE Healthcare Bio-Sciences AB) density gradient centrifugation. For xCELLigence assay, NK cells were purified by negative immune selection using the NK selection kit (Miltenyi Biotech), following company instructions. Purified NK cells (0.5–1.6 × 10^6^/ml) were cultured in RPMI 1640 medium (GIBCO Invitrogen) supplemented with IL-2 (1000 IU/ml; Miltenyi Biotech) and 10% human serum AB (Biowest) for 2 days.

### Cell Lines

The colon carcinoma cell line DLD-1 (ATCC) was maintained in Dulbecco’s modified eagle medium (DMEM, Invitrogen) supplemented with 10% heat-inactivated fetal calf serum (FCS) Natocor, 2 mM l-glutamine, 3.5 mg/ml sodium bicarbonate, 4.5 mg/ml glucose, and 1% Penicillin–Streptomycin (Invitrogen). The leukemic cell line K562 (ATCC) was maintained in RPMI 1640 supplemented with 10% FCS and 1% Penicillin–Streptomycin.

### CD107a Degranulation and IFN-γ Secretion Assays

Approximately 10^6^ PBMC were cultured at 10:1 effector/target (E:T) ratio for 6 h at 37°C with K562 cells and incubated with anti-CD107a-FITC. After 1 h, protein transport inhibitor (Golgi Stop-BD) was added. Five hours later, cells were labeled in PBS for 30 min at 4°C with anti-CD56-APC and anti-CD3-PerCP, after that, cells were fixed and permeabilized (Cytofix/Cytoperm, BD Biosciences) and washed (Perm/Wash, BD Biosciences). Finally, cells were labeled in Perm/Wash buffer for 30 min at 4°C with anti-IFN-γ-PE (BD Biosciences) and then collected on a FACSCalibur flow cytometer. The results are expressed as the percentage of IFN-γ^+^ or CD107a^+^ gated on NK cells. Spontaneous basal IFN-γ secretion and degranulation were determined in absence of targets and cytokines.

### Lysis and ADCC Experiments

DLD-1 cells were used as target and labeled with Calcein-acetyoxymethyl (Calcein-AM; Molecular Probes, Invitrogen Life Technology). The effector cells were PBMC, normalized by percentage of NK cells. The cytotoxicity assay was performed at 2.5:1 E:T ratio, in triplicate, with 1 μg/ml of cetuximab or control mAb (rituximab). Three replicate wells for spontaneous (only target cells in RPMI medium with 10% FCS) and maximum release (only target cells in medium plus 1% Triton X-100) were measured. After incubation at 37°C in 5%CO_2_ for 4 h, supernatants were analyzed by fluorimetry (OPTIMA-BMG Labtech) to measure cell death (Calcein release), and the percentage of specific lysis was calculated as: (experimental fluorescence−spontaneous fluorescence)/(maximum fluorescence−spontaneous fluorescence) × 100.

### xCELLigence Assays

Natural killer cell-mediated lysis of DLD-1 cells was assessed by xCELLigence System (Roche), a label-free real-time monitoring assay of adherent cell lysis (26). It measures electrical impedance across interdigitated micro-electrodes integrated on the bottom of tissue culture E-Plates. The electrode impedance, which is recorded as a dimensionless parameter denominated cell index (CI) value, provides quantitative information about the status of the adherent cells, including cell number, viability, and morphology. DLD-1 targets (15,000 cells) were seeded into the wells of 96 E-Plates in 100 μl of RPMI 1640 medium plus 10% human serum and their adhesion was monitored for 5 h. IL-2-activated purified NK cells were added in a volume of 50 μl/well at 1:1 E:T ratio. Cocultures were assessed by the system with a measurement of CI every 15 min for up to 300 min. Results expressed as CI, were normalized with RTCA Software (Roche, Applied Science, Mannheim, Germany) (nCI) and are expressed as % specific lysis:% lysis = (nCI (no effector) − nCI (effector))/nCi (no effector) × 100. In some cocultures, NK cells were pretreated with anti-NKp46, anti-NKp30, anti-NKG2D, or anti-DNAM-1 mAbs (R&D System).

### Flow Cytometry

Direct immunofluorescence was performed on PBMC or NK cells using specific mAbs and isotype-matched mAbs. Cells were washed with PBS and stained for 30 min at 4°C. NK cells, gated on CD3^−^CD56^+^ cells in the lymphocyte FSC/SSC subset were analyzed for the expression of CD16, NKp46, NKp30, NKp44, NKG2D, DNAM-1, CD161, CD94, CD8, NKG2A, CD85j, CD158a, and CD158b (PE-conjugated antibodies, BD Biosciences). mAbs and respective clones are listed in Table S1 in Supplementary Material. Cells were collected on a FACSCalibur flow cytometer (BD Biosciences). These data were analyzed using FlowJo software.

### ELISA

Plasma samples were tested for the presence of HLA-E and HLA-G soluble molecules by home-made ELISA tests. TGF-β content in human plasma was evaluated using a human TGF-β 1 ELISA kit (BD) following the manufacturer’s instructions.

### Cytokines and Lenalidomide Treatment

Natural killer cells were incubated overnight in medium supplemented with rhIL-2 1000 IU/ml, rhIL-15 10 ng/ml, or lenalidomide 10 μM (LC Laboratories) prior to performing degranulation, IFN-γ release, lysis, and ADCC, as described above.

### Statistical Analysis

Comparisons between CRC patients and HD groups were assessed by non-parametric Mann–Whitney test when samples did not present normal distribution or parametric *t*-test, when samples presented normal distribution. For functional assays, we used paired *t*-test to compare different *in vitro* treatment conditions. Correlations were analyzed by Pearson (when samples presented normal distribution) or Spearman tests (in case of samples not normally distributed). The survival curves were plotted according to the Kaplan–Meier method and compared using the Log-rank test (*p* values <0.05 noted as *, *p* < 0.01 as ** and *p* < 0.001 as ***).

## Results

### CRC Patients Showed Altered Proportions of NK Cells and Phenotypic Abnormalities

**Figure 1:**
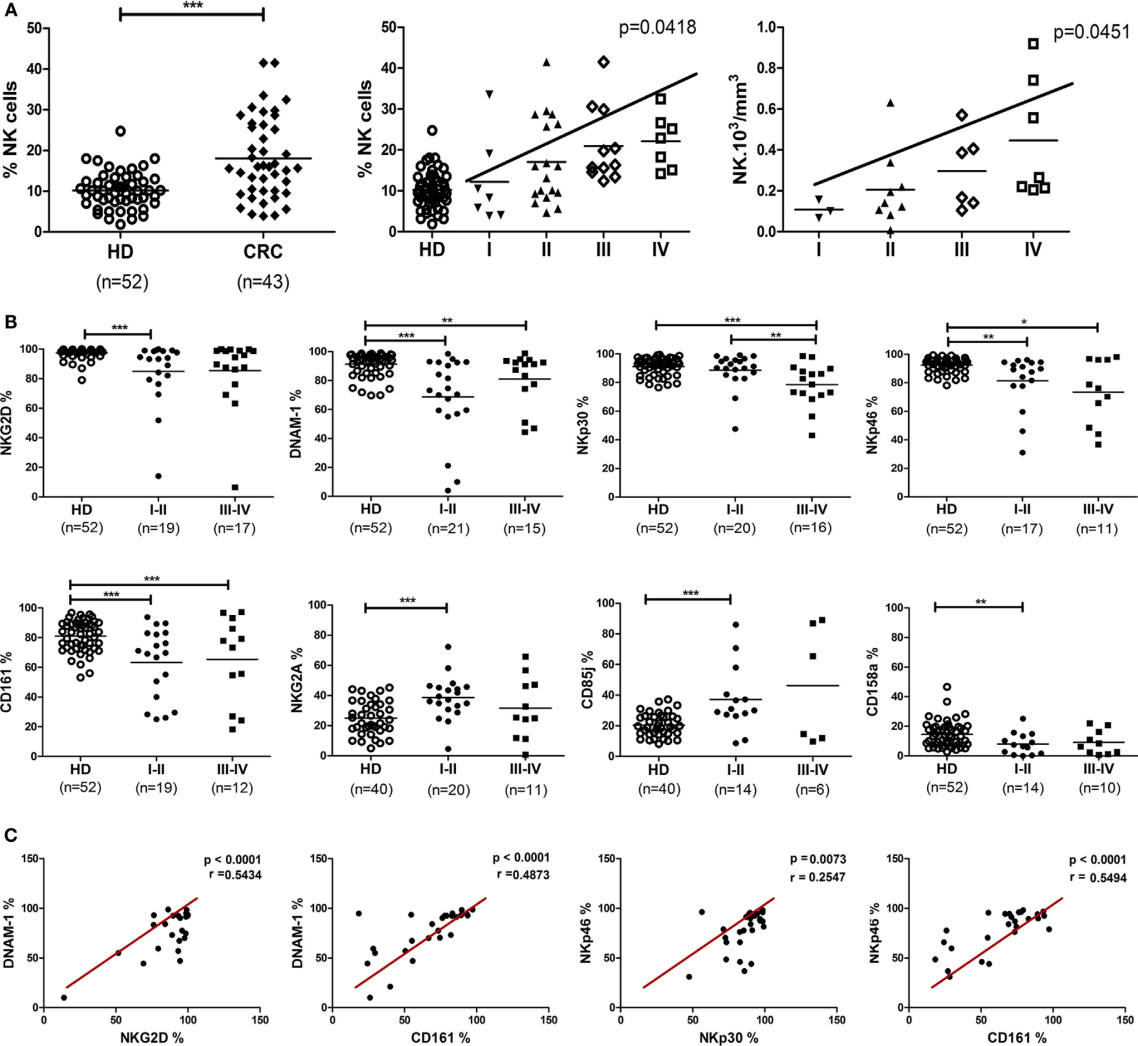
**Proportions, absolute numbers, and phenotype of peripheral blood NK cells from CRC patients**. **(A)** *Left*: NK proportions in lymphocytes population of CRC patients (*n* = 43) and healthy donors (*n* = 52). Each symbol corresponds to different samples. For statistical analysis unpaired *t*-test was used. *Middle*: CRC-NK proportions in patients at different stages of the disease. Linear trend by one-way ANOVA test (*p* < 0.05). *Right*: distribution of CRC-NK absolute numbers expressed as 10^3^/mm^3^ (*n* = 25). Linear trend by one-way ANOVA test (*p* < 0.05). **(B)** Expression of NK cell receptors. Activating and inhibitory receptor expression by peripheral blood NK cells from CRC patients of early (I–II) and late (III–IV) stages compared with healthy donors. For statistical analysis unpaired *t*-test or Mann–Whitney test were used. Horizontal bar represents mean value. *p < 0.05; **p < 0.01; ***p < 0.001. **(C)** Positive correlations between activating receptor expression in peripheral blood NK cells from CRC patients. Correlations between different receptor expressions were performed with Spearman or Pearson test. Regression coefficients *r* and *p* values on each graph.

In an observational and exploratory study, 52 CRC patients were enrolled, 34 from I–II stages without lymph node invasion and 18 from stages III–IV with lymph node or metastatic dissemination. Fifty-two HD were analyzed as reference. The clinical characteristics of patients are summarized in Table 1. Phenotypic analysis from CRC patients (43/52) displayed a significant increase of PB CRC-NK cell proportions among the lymphocyte subset as compared with HD (*p* < 0.001) (Figure 1A, left panel). Since mean age was significantly higher in the group of CRC patients (61.4 ± 12.0 vs. 38.0 ± 13.0 in HD, *p* < 0.0001), a linear regression model was developed using the percentage of NK cells as the outcome variable, and the presence of cancer and age as regressive variables. The presence of cancer increases by 5.29% the amount of NK cells after adjusting by age (IC95 = 1.004–9.579, *p* = 0,016). Surprisingly, CRC-NK percentages were increased from stage II, reaching progressively the highest values in stage IV (linear trend ANOVA, *p* = 0.0122) (Figure 1A, middle panel). We also found higher absolute numbers of PB CRC-NK cells from available blood cell counts prior to surgery (25/43) (Figure 1A, right panel). CD56^dim^ percentages in CRC-NK cells were similar to HD-NK cells (%CD56^dim^ CRC-NK 93.06 ± 6.24 *N* = 43 vs. %CD56^dim^ HD-NK 91.48 ± 5.01 *N* = 52). We, then, studied NK cell *ex vivo* phenotype according to the gating strategy depicted in Figure S1 in Supplementary Material. Most of the 13 NK cell receptors analyzed showed high inter-individual variability in CRC patients. Moreover, eight of these receptors presented modified expression in CRC patients compared with donors NK cells. Furthermore, the altered phenotypic expression was evident in CRC-NK from early stages of the disease (Figure 1B). The percentages of most activating receptors, NKG2D, DNAM-1, CD161, and NKp46 were decreased from non-metastatic stages (*p* < 0.001, *p* < 0.001, *p* < 0.001; and *p* < 0.01, respectively), while NKp30 presented diminished expression only in advanced ones (*p* < 0.001). The inhibitory receptors, CD85j and NKG2A were augmented from stages I–II (*p* < 0.001), while the KIRs 2DL1/2DS1 recognized by anti-CD158a (27) was also diminished in early stages (*p* < 0.01). Expression of CD16, NKp44, CD94, CD158b (that encompasses KIR2DL2/2DL3/2DS2) (27), and CD8 were comparable to values observed in HD-NK cells (data not shown). Significant positive correlations were found between different activating receptors, including NKG2D, DNAM-1, NKp30, NKp46, and CD161, confirming the broadly phenotypic imbalance in CRC-NK (Figure 1C). In HD-NK cells, activating receptors displayed high and homogeneous percentages that limited correlations mainly to the NCR group and CD16 (data not shown).

Based on the association between NK cell percentages and clinical stage, we investigated the involvement of other clinical features in NK cell receptor expression, as displayed by CRC patients. NK cells from patients with poorly differentiated tumors, considered of bad prognosis, highly expressed NKG2D and DNAM-1 activating receptors (*p* < 0.05 in both cases) and also higher expression of CD94 (*p* < 0.05). This receptor, which conforms the heterodimer CD94-NKG2A or CD94-NKG2C, was also regularly expressed in CRC-NK from patients with elevated carcino-embryonic antigen (CEA) values, associated as well with poor prognosis. Moreover, NKG2A and CD8 expression were diminished (*p* < 0.05 and *p* < 0.01, respectively) in patients with positive lymphatic vascular invasion (LVI), that is a strong bad prognostic marker and taken into account in treatment determination for stage II–III patients (Table S2 in Supplementary Material).

### CRC-NK Exert Defective Functionality

**Figure 2:**
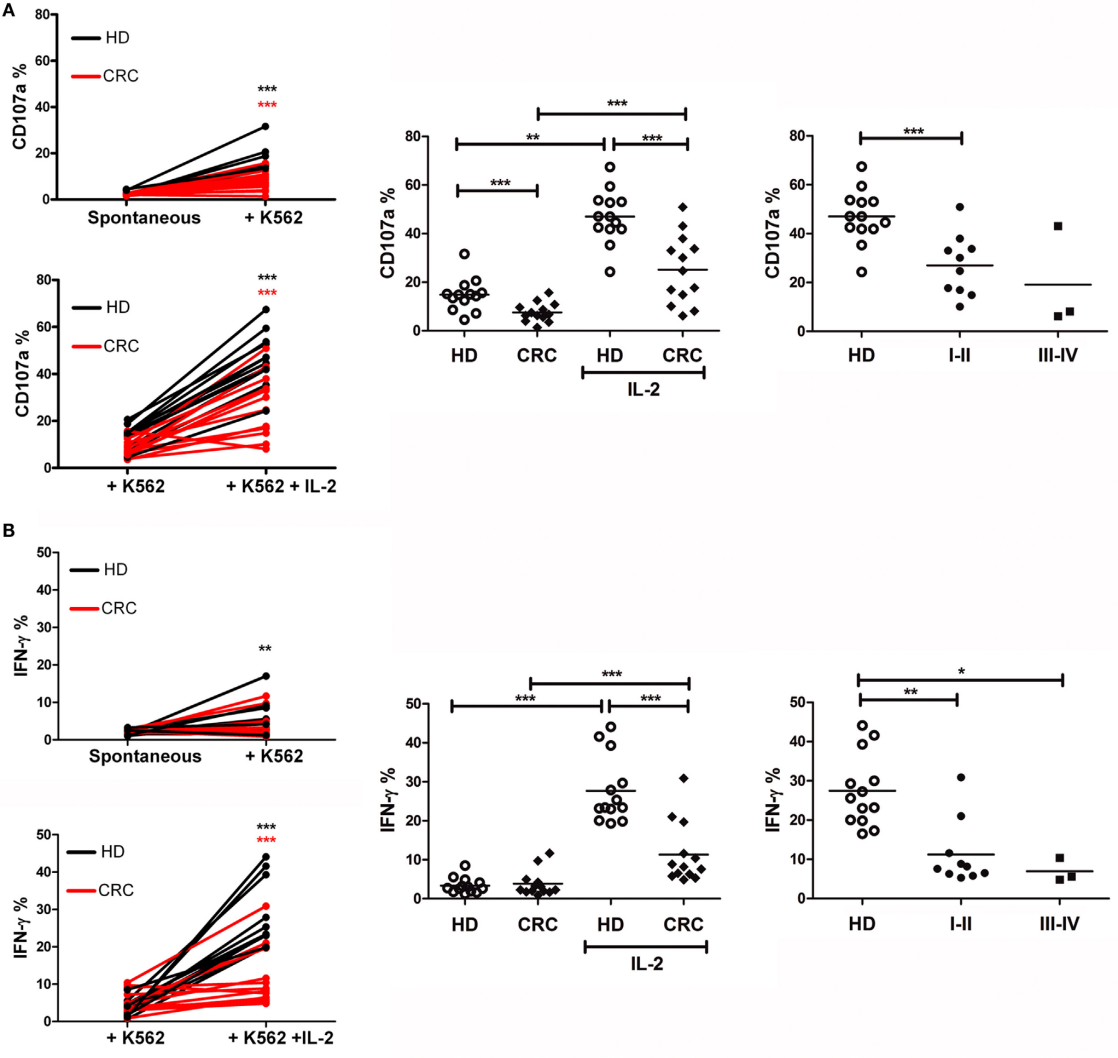
**Peripheral blood CRC-NK cells exhibit defective functionality**. **(A)** NK cell degranulation capacity. *Left*: degranulation by HD (black) and CRC patient (red) NK cells stimulated by K562 with (lower) or without IL-2 pre-activation (upper). Comparisons were analyzed by paired *t*-test. *Middle*: %CD107a^+^ NK cells from HD and CRC patients pre-stimulated or not with IL-2 (*n* = 13). *Right*: degranulation by IL-2 pre-stimulated NK cells from HD or CRC patients according to early (I–II) and late (III–IV) stages. Comparison of NK cell function between donors and patients were analyzed by non-parametric Mann–Whitney test. **(B)** IFN-γ production by NK cells. *Left*: IFN-γ secretion by HD-NK (black) and CRC-NK cells (red) NK cells stimulated by K562 with (lower) or without IL-2 pre-activation (upper). Comparisons were analyzed by paired *t*-test. *Middle*: percentages of IFN-γ^+^ NK cells from HD and CRC patients, pre-stimulated or not with IL-2 (*n* = 13). *Right*: IL-2-pre-stimulated NK cells from HD and CRC patients according to early (I–II) and late (III–IV) stages. Comparison of NK cell function between donors and patients were analyzed by non-parametric Mann–Whitney test. Horizontal bar represents mean value. *p < 0.05; **p < 0.01; ***p < 0.001.

Based on the phenotypic alterations of CRC-NK cells, we assayed the NK cell degranulation potential (CD107a surface expression) and IFN-γ production in response to challenge by K562 cells as canonical target. Freshly isolated PBMC, either incubated in medium or stimulated overnight with IL-2, were then cultivated with K562 cells for 6 h before analysis by flow cytometry (see Materials and Methods). Challenge with K562 cells led to CD107a expression in HD-NK cells, while NK cells from several patients kept their values close to spontaneous degranulation (Figure 2A, left panel). IL-2 pre-stimulated NK cells from HD and patients exhibited significantly augmented responsiveness (*p* < 0.001 in both cases). Still, CD107a expression levels of CRC-NK cells were lower than those achieved by HD-NK cells (*p* < 0.001 for both un-stimulated and stimulated NK cells) (Figure 2A, middle panel). Corresponding to phenotype alterations, diminished degranulation activity was detected in CRC patients at stages I–II showing an early immune impact on NK cells (Figure 2A, right panel).

Natural killer cells’ antitumor effects also implicate the production of cytokines, such as IFN-γ. In this regard, culture with K562 did not induce significant levels of IFN-γ production on resting NK cells (Figure 2B, left panel). IL-2-pre-stimulation enhanced significantly the levels of IFN-γ production by HD-NK and in a lower extent by CRC-NK cells (*p* < 0.001) (Figure 2B, left and middle panel). Once again, the low functional capacity was observed since early stages of disease (Figure 2B, right panel).

### Lytic Activity against CRC Cells Is Reduced in CRC-NK

**Figure 3:**
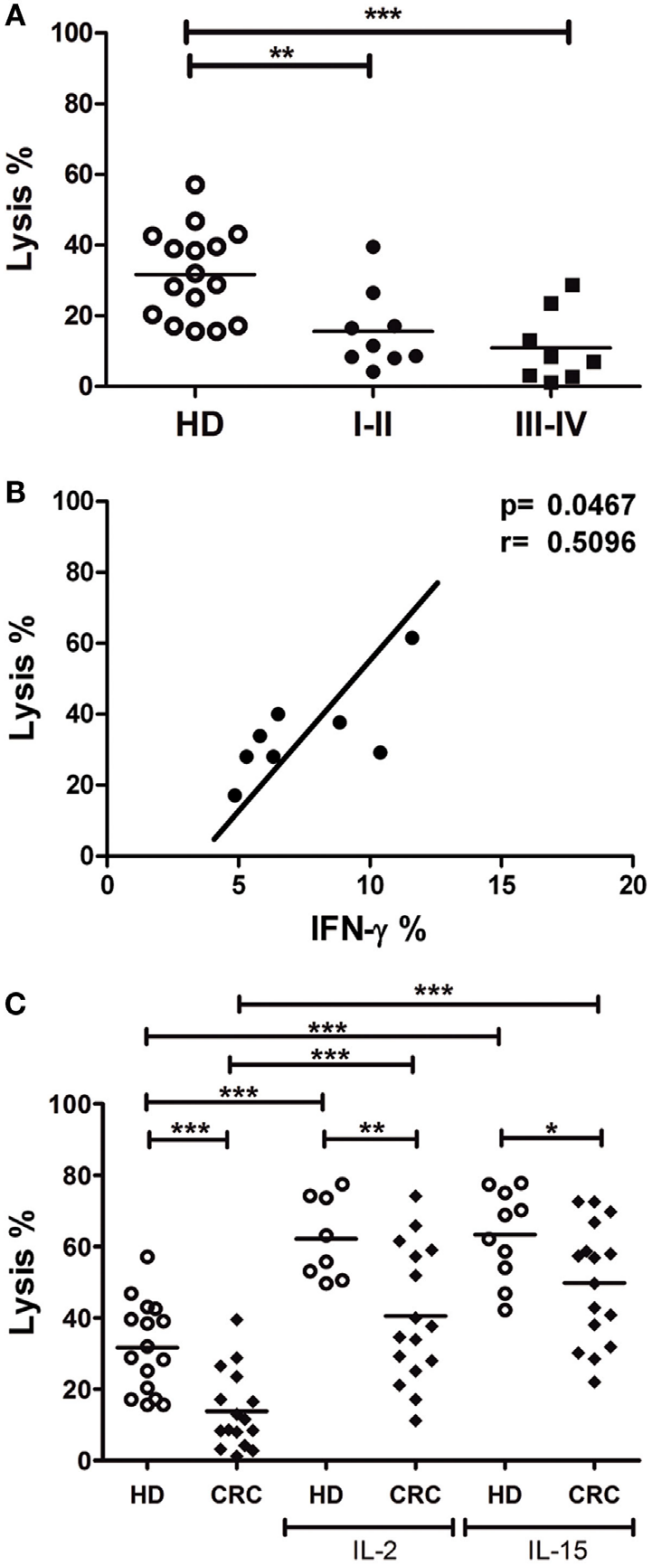
**Lytic activity against CRC cells is reduced in CRC-NK cells**. **(A)** Lysis percentage by CRC-NK (*n* = 17) and HD-NK (*n* = 16) cells measured by calcein release test. DLD-1 tumor cells were used as target at an E:T ratio of 2.5:1. Patients were divided in early (I–II) or late (III–IV) stages. **(B)** Positive correlation between functional activities of NK cells from CRC patients, lysis capacity, and IFN-γ production. **(C)** Lysis percentage of DLD-1 cells by CRC-NK and HD-NK pre-stimulated by IL-2 or IL-15. Horizontal bar represents mean value. *p < 0.05; **p < 0.01; ***p < 0.001.

In order to evaluate NK cell cytotoxic capacity against CRC cells, we performed lysis assays of CRC cell line DLD-1 by HD and CRC-NK cells, using colorimetric calcein release test. CRC-NK also presented lower lytic capacity against DLD-1 since early stages of disease (Figure 3A), as we could anticipate from degranulation experiments (Figure 2). In addition, we observed an association between NK cell function, IFN-γ production, and lysis capacity in response to DLD-1 stimulation (*p* < 0.05) (Figure 3B). Even though IL-2- or IL-15-activated CRC-NK cells improved their lytic activity against CRC cells, they could still not reach HD-NK lytic ability (Figure 3C).

### NKp46 and NKp30 Expression Is Involved in Reduced CRC-NK Functionality

**Figure 4:**
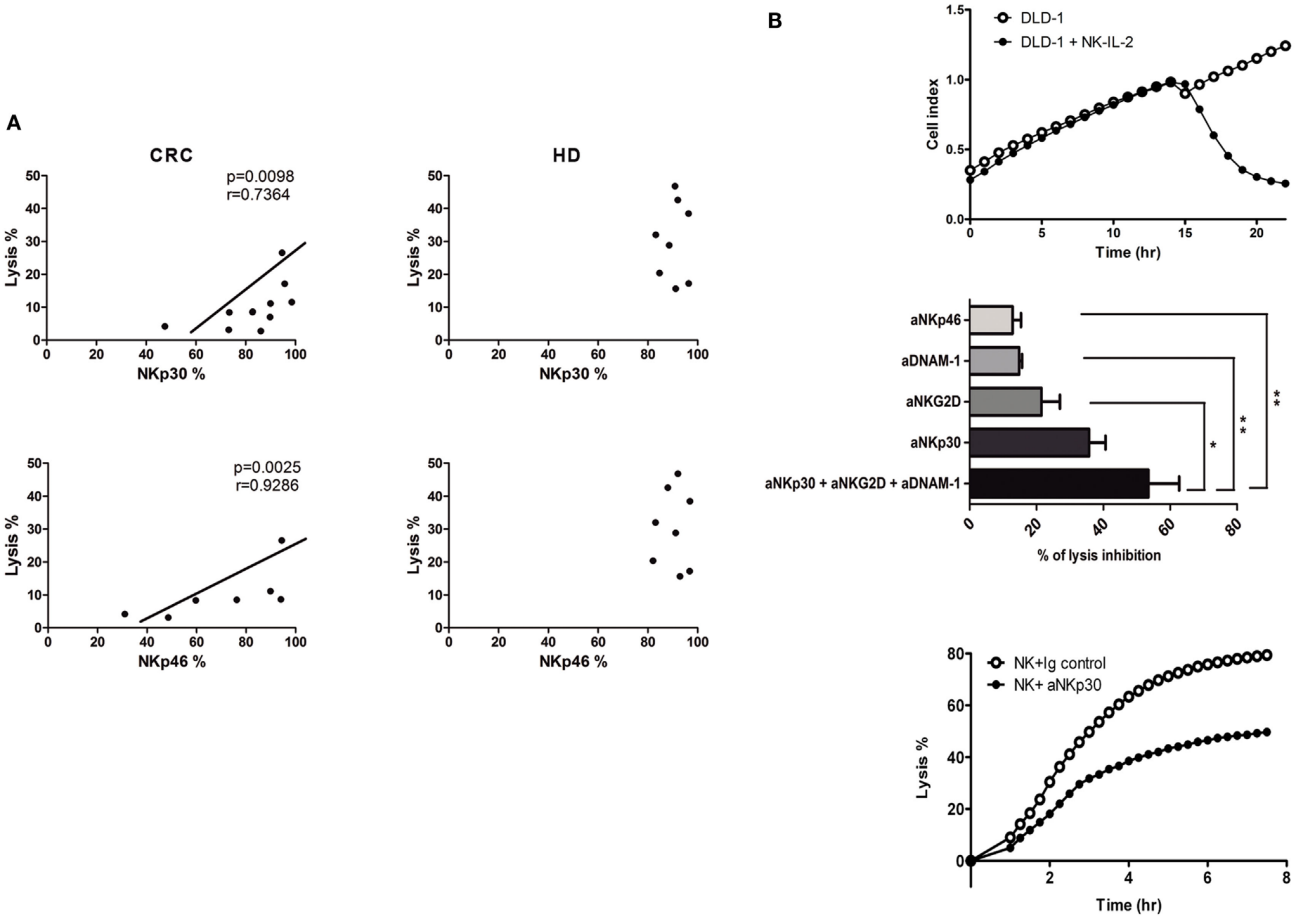
**NK cell receptors involved in lytic activity**. **(A)** Correlations between NK cell receptor expression and lysis activity against DLD-1 cells from CRC-NK (left) and HD-NK (right). Correlations were performed with Spearman or Pearson test. Regression coefficients *r* and *p* values on each graph. **(B)** *Upper*: representative graph of dynamic measurement of DLD-1 cell index vs. time in control conditions (white dots) or with IL-2-activated NK cells from HD (black dots). *Middle*: percentage of DLD-1 cells lysis inhibition using different blocking antibodies against NK cell-activating receptors measured at 300 min (*n* = 3 independent experiments). Percentage of lysis inhibition was calculated as: (% lysis with isotypic control mAb − % lysis with blocking mAb)/% lysis with isotypic control mAb × 100. Comparison was performed using one-way ANOVA. *Lower*: percentage of DLD-1 lysis by IL-2-activated HD-NK cells with isotype mAb control or NKp30 blocking mAb. E:T 1:1 NK: DLD-1. *p < 0.05. **p < 0.01.

Correlation analyses were performed in order to establish which of the receptor abnormalities in CRC-NK play a major impact on the low-lytic activity against CRC cells. Expression levels of activating NCR NKp30 and NKp46, were positively correlated with DLD-1 cells lysis (*r* = 0.7364, *p* = 0.098; *r* = 0.9286, *p* = 0.0025; respectively) (Figure 4A). These associations were not present in HD-NK, mainly because HD exhibit homogeneous high expression of activating receptors and homogeneous low expression of the inhibitory ones (Figure 1B).

To elucidate the impact of these receptors in CRC cells cytotoxicity, we assessed the lysis of DLD-1 cells by purified IL-2-activated HD-NK cells using a dynamic assay that quantifies the CI of adherent targets. The lytic activity of NK cells provoked the detachment/apoptosis of CRC cells, as evidenced by an abrupt decay of CI after the addition of effectors (Figure 4B, upper panel). From the CI values, lysis curves were calculated. In some experiments, receptor-neutralizing mAbs, previously tested to block NKp46, NKp30, NKG2D, and DNAM-1 receptors in melanoma (28) and renal carcinoma (29) were used (Figure 4B, middle panel). Separate addition of anti-NKp46, anti-DNAM-1, or anti-NKG2D mAbs decreased CRC cell lysis; the addition of anti-NKp30 mAb induced the highest percentage of inhibition (35.70 ± 4.95%). Addition of anti-DNAM-1 and anti-NKG2D mAbs slightly synergized when combined with anti-NKp30 (Figure 4B, middle panel). These data indicate that the lysis of DLD-1 cells by IL-2-activated NK cells is mediated mainly by NKp30 (Figure 4B, lower panel) with the contribution of NKp46, NKG2D, and DNAM-1.

### Soluble TGF-β Is Augmented in CRC Patients

**Figure 5:**
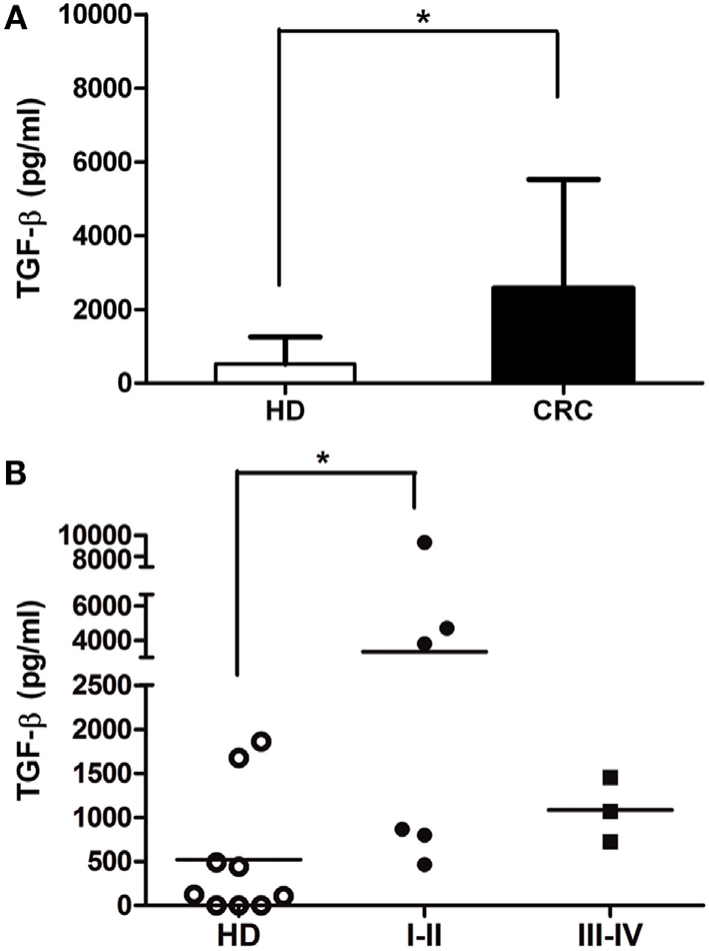
**Soluble TGF-β is augmented in CRC plasma**. **(A)** TGF-β concentration (picograms per milliliter) in plasma of CRC patients and HD, measured by ELISA (*n* = 9). **(B)** TGF-β levels of CRC patients according to disease stage (I–II and III–IV). Horizontal bar represents mean value.* *p* < 0.05.

Several soluble molecules were associated with receptor expression modulation and the impairment of NK cell activity (30, 31). To determine whether CRC patients presented augmentation of NK cell suppressive molecules, we performed ELISA assays from plasma patients. Soluble HLA-E molecules (>5 pg/ml) were detected in 7/35 patients (mean 22.9 ± 53.4; range 0–187.4 pg/ml), and soluble HLA-G molecules (>5 ng/ml) were detected in the plasma of 6/43 patients (mean 1.8 ± 5.1; range 0–23.0 ng/ml). Non-significant differences were found as compared with plasma samples from HD (data not shown). However, the amount of total circulating TGF-β in the plasma of CRC patients, both early and late stages, was almost fivefold higher than in HD (Figure 5). Even when analyzed samples belonged to other CRC patient than those analyzed in functional assays, the increment of TGF-β production by CRC tumors could impact on NK cell phenotype and their respective functionality (32).

### NKp46 Expression Is Correlated with Relapse-Free Survival of CRC Patients

**Figure 6:**
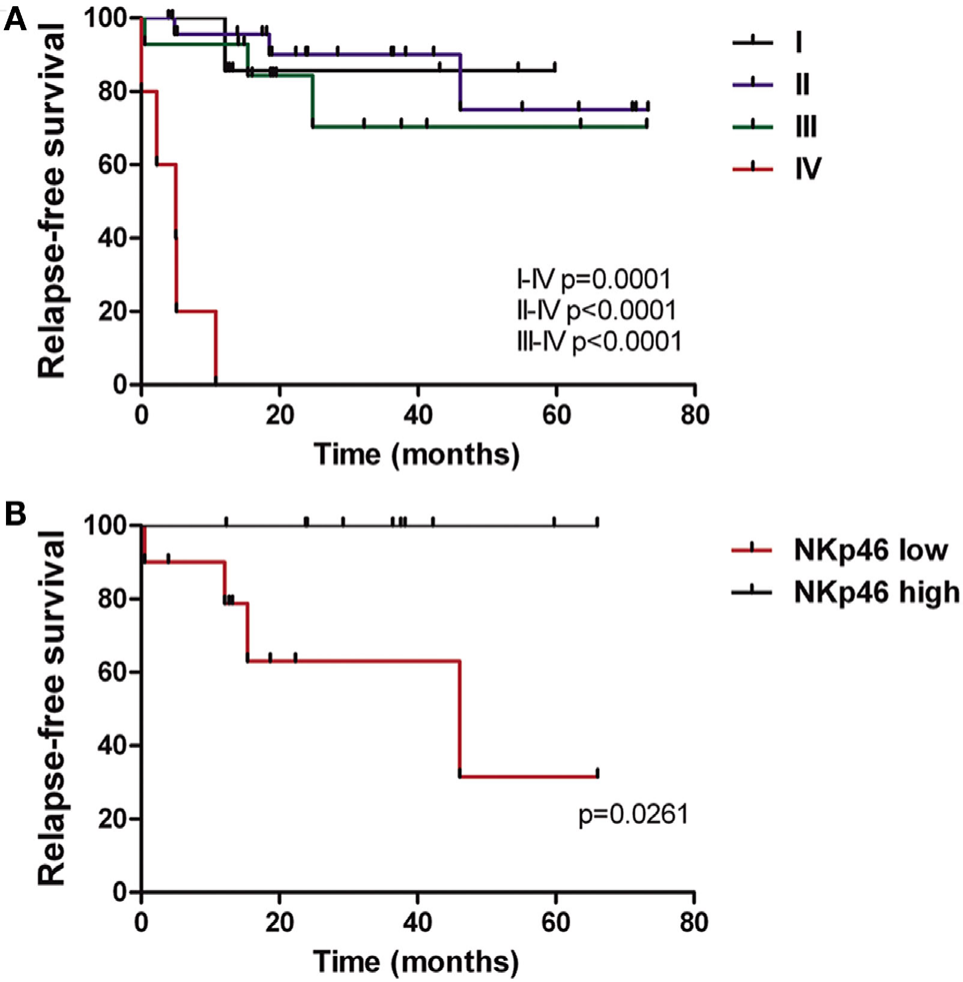
**NKp46 expression is correlated with relapse-free survival of CRC patients**. **(A)** Kaplan–Meier curves of CRC patients (*n* = 52) performed as relapse-free survival percentage through time according to disease stage (I–IV). **(B)** Relapse-free survival curves of CRC patients (*n* = 10 in each group) according to NKp46 receptor expression by NK cells. Median value of NKp46 percentage in CRC population (86.9%) was used as cut-off between the two groups.

Taking into account that deregulation of NK cell receptor expression was associated with function detriment, we analyzed if the exhausted phenotype had a correlation with clinical outcomes in a retrospective study. Patients were followed for a median of 24.7 months and during this period 11/52 (21%) relapsed. As expected, clinical staging IV at diagnosis impacted on RFS (Figure 6A), while stage I, II, and III outcomes did not differ significantly. Considering that several receptors were underexpressed since early stages, we evaluated their expression in stages I, II, and III population (with the exclusion of stage IV patients) and correlate it with RFS. The patients were divided into groups of low and high NK cell receptor expression, using the median percentage value of each receptor as a cut-off. We found that expression of NKp46-activating receptor impacted on RFS by significant dissociation of Kaplan–Meier curves (*p* = 0.0261) (Figure 6B). Within stage IV, 100% (5/5) of patients progressed, independently of NKp46 status; nevertheless, it could be further analyzed if receptor expression had relevance in progression-free survival (PFS). Our data indicate that NKp46 inference warrants more investigation and validation with a larger cohort as well as longer follow-up, as it could have prognostic implications.

### CRC-NK Functionality Can Be Stimulated by Immune Therapies

**Figure 7:**
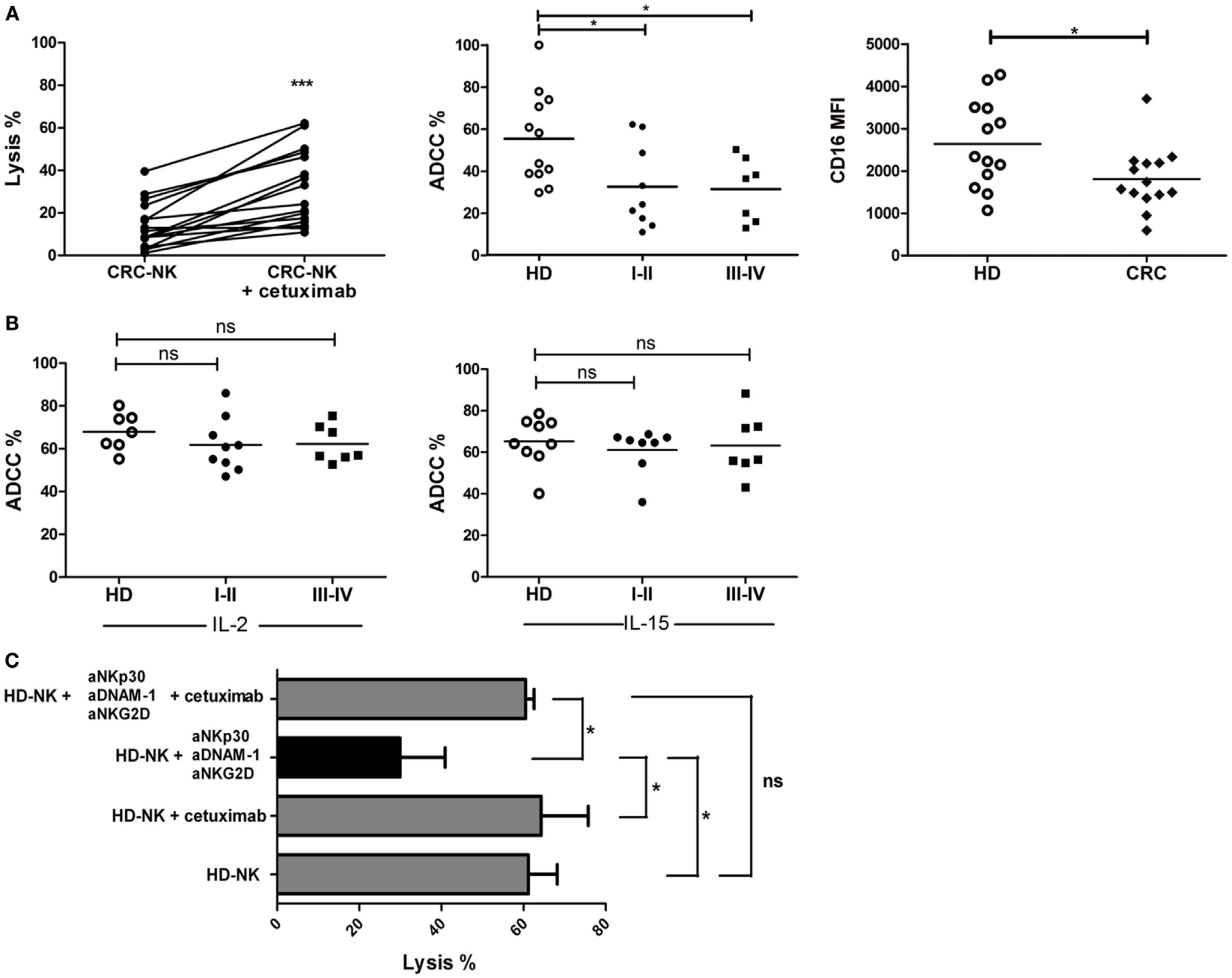
**CRC-NK lysis is restored by cetuximab**. **(A)** *Left*: percentage of DLD-1 lysis by CRC-NK cells at 2.5:1 E:T ratio assessed by calcein release test in presence of an isotype control mAb or Cetuximab (1 μg/ml). *Middle*: ADCC percentages of CRC patients (*n* = 16) divided in early (I–II) and late (III–IV) stages. *Right*: CD16 mean fluorescence intensity (MFI) measured in CRC-NK (*n* = 14) and HD-NK cells. **(B)** Percentages of DLD-1 cells lysis at 2.5:1 ratio in presence of cetuximab (1 μg/ml) by IL-2- (left) or IL-15- (right) pre-stimulated CRC-NK and HD-NK cells. Patients were divided in early (I–II) and late (III–IV) stages. **(C)** Percentages of DLD-1 lysis by IL-2-pre-stimulated purified HD-NK cells in presence of isotype antibodies; cetuximab; NKp30, DNAM-1, and NKG2D blocking antibodies; or these blocking antibodies plus cetuximab (*n* = 3 independent experiments). *p < 0.05; ns, non-significant.

To identify immune strategies to restore CRC-NK functionality, we addressed different *in vitro* treatments, including the pre-incubation of tumor cells with cetuximab and NK cell stimulation with lenalidomide, IL-2, or IL-15. Pre-treatment of DLD-1 cells with cetuximab significantly augmented tumor cell lysis mediated by CRC-NK (Figure 7A, left panel). Still, ADCC response of CRC-NK cells reached lower levels than HD-NK, in both early and late stages (Figure 7A, middle panel), probably due to diminished molecule density of CD16 (FcγR) in CRC-NK (Figure 7A, right panel). However, addition of IL-2 or IL-15 to NK cells completely restored ADCC capacity to HD-NK levels (Figure 7B). In this respect, cetuximab combined with cytokines allowed CRC-NK to reach normal lytic activity. Pre-stimulation with IL-2 or IL-15 modulated several NK cell receptors that could contribute to enhance functionality (Figure S2 and Table S3 in Supplementary Material). Lenalidomide pre-treatment did not improve NK cell activity (data not shown). Finally, we performed a dynamic lysis assay in which low lytic activity due to DNAM-1, NKG2D, and NKp30 blockade was reestablished by cetuximab combined with IL-2 pre-stimulation (Figure 7C). Cetuximab itself did not have any effect on the CI of DLD-1 cells due to their KRAS mutational status (data not shown); the antibody acted only through the immune pathway. We observed a greater relative increase in functionality of CRC-NK than HD-NK cells in response to cytokines and mAb joint stimulus (almost fourfold higher), while the relative increase in response to independent activation with cytokines or cetuximab did not differ between CRC patients and HD (Figure S3 in Supplementary Material).

## Discussion

Natural killer cells are major cytotoxic innate immunity players against tumor cells in the early immune defense, monitoring aberrant ligand expression that usually occurs during malignant transformation. The ability of NK cells to rapidly lyse target cells without previous immunization could be significant in tumor restraint, and their cytokine release may have a strong impact on adaptive immunity. Several approaches have recently been developed to enhance NK cell antitumor function to prolong *in vivo* survival (after adoptive cell transfer therapy) and promote “homing” to the tumor microenvironment, potentially leading to improvement in therapeutic outcome (33). Therefore, we performed an extensive study of NK cells in CRC patients, with the aim of finding a rationale for the development of combined NK-based immunotherapies with current treatments. Given that colorectal tumor-infiltrating NK cells are scarce and deeply deregulated (21, 34), they do not seem to exert a relevant activity at tumor site. Nevertheless, circulating NK cells may display a role in metastasis control as it has been recently demonstrated (35). Impairment of NK cell activity in blood circulation has been identified in several human cancers, including breast cancer and melanoma, mainly due to defective phenotypic balance between activating and inhibitory receptors (36–38). These phenotypic alterations exert a negative effect on NK cell functionality and the NCR group seems to be the principal regulating pathway (39). In particular, Fregni et al. (28) determined that low expression of NKp46 in PB NK cells of stage IV melanoma patients was associated with a faster progression.

In this study, we report that NK cells were more abundant in PB of CRC patients than in HD, reaching higher values in stage IV. This was a surprising finding given that the number of circulating NK cells in other malignancies was not found to differ from the normal population (28, 39). However, higher percentages and number of NK cells did not correspond to increased antitumor activity, as CRC-NK cells displayed an altered expression of the main activating receptors. While in most cancers, NK cell phenotypic and functional dysregulation was observed in tumor-infiltrating NK cells (40–45), only a few PB-NK cells receptors were found altered (28, 36, 40, 41). However, CRC-NK cells presented a profound disequilibrium with underexpression of CD16, NKG2D, DNAM-1, NKp30, NKp46, CD161, and CD158a and overexpression of the inhibitory receptors CD85j and NKG2A. By blocking these receptors in dynamic lysis assays; here, we provide evidence for the potential linkage between NKp30, NKG2D, and DNAM-1 and NK activity against CRC cells. It was previously demonstrated that NCRs and NKG2D can trigger a complementary or synergistic response (46); however, in our *in-vitro* setting, NKp30 was the pivotal receptor using DLD-1 cells as target. This deregulated phenotype and consequent functional detriment was present in all clinical stages of disease implying an early remodeling of innate immune subsets that could favor tumor progression. Meanwhile patients with poorly differentiated tumors, which generally indicate a worse outcome, displayed higher percentages of NKG2D and DNAM-1 perhaps due to tumor additional protection of mucin and alternative mechanisms of immune evasion. Conversely, Peng et al. (22) observed that the percentage of NKG2D positively correlated with histological grade in CRC (*p* < 0.01), and they observed NKp46 downregulation in stage IV. None of the molecules studied in that research were associated with blood vessel invasion or nerve invasion in CRC. In contrast, we found an association between low CD8 expression in NK cell population and tumor vascular invasion. It has been suggested that NK cells expressing CD8 maintain greater lytic activity as proposed that this subset of NK cells could potentially be associated with this clinical feature (47, 48).

Colorectal cancer-NK cells challenged with tumor cells presented decreased IFN-γ production, degranulation, and lysis in all stages of disease. This observation contrasts with findings in melanoma, lung, and breast cancer patients (28, 36, 38); for whom NK cell alterations were higher in advanced metastatic patients. Given that downregulation of activating receptors and upregulation of the inhibitory receptors are both present in early stages, it is possible that low NK cell functionality prevents NK cells from efficiently attacking and eliminating tumor cells in circulation at premature and advanced phases of disease. In this regard, it would be interesting to evaluate pre-neoplastic colorectal lesions, in order to test the hypothesis that an immune system characterized by dysregulated NK cells could be a substrate for tumor establishment.

In an exploratory and observational cohort, we demonstrated that patients whose NK cells expressed high values of NKp46 at surgery had statistically longer RFS compared with patients presenting low NKp46. This receptor was previously associated with progression in stage IV melanoma patients (28). Pagès et al. showed that *in-situ* immune infiltrate was a positive prognostic factor for disease evolution and survival (8). Surprisingly, in our study, NKp46 was associated with longer RFS in stages I to III CRC. More interestingly, this difference continued to be statistically significant when stages II and III were analyzed independently (data not shown). Of note, most patients in our cohort received adjuvant treatment after surgery, many of them probably unnecessarily either because they were already cured or because they will relapse despite treatment. Currently, clinical and pathological variables of disease cannot predict prognosis, it is essential to identify patients who will benefit from adjuvant therapy; hence, we are planning to confirm these findings in a larger, prospective, observational study.

Natural killer cell deregulation may be a consequence of soluble factors produced by tumors that promote a systemic inhibition without direct contact to the immune cells (31, 49–55). TGF-β exerts numerous effects on NK cells, including the inhibition of cytokine secretion, proliferation, cytotoxicity, and the downregulation of activating receptors, such as NKG2D and NKp30 (52, 53, 55, 56). TGF-β levels often elevated in the sera of cancer patients were associated with inhibition of immune function, including weakened NK cell responses, and poor prognosis (45, 54, 55). Therefore, the augmentation of plasma TGF-β observed in a few patients could be responsible for the major dysregulation in CRC-NK from early stages of the disease. Further analyses in a higher number of samples are needed to better study TGF-β expression and phenotypic/functional alteration in CRC-NK cells.

In an effort to reach an efficient NK cell status, a combination of therapies from early stages of disease could be developed, such as stimulation by cytokines and administration of mAbs to facilitate ADCC of NK cells. We did not observe any potentiating effect by lenalidomide *in vitro*, an immune-stimulating drug reported to activate T lymphocytes to secrete IL-2 that would, at the same time, stimulate NK cells (57). However, it would be important to study the effect of lenalidomide in *in-vivo* models.

By targeting EGFR with the antibody cetuximab, administered to treat metastatic K-RAS wild-type CRC patients (58), our data indicate that exhausted NK cells could be activated in presence of IL-2 or IL-15. Currently, several clinical trials are evaluating the uses of mAbs and cytokines (NCT02507154, NCT00569296 and NCT00625729 among others; https://clinicaltrials.gov). An alternative therapy that would be interesting to test is the administration of anti-TGF-β antibodies, in combination with cetuximab and cytokines, to eliminate a possible pathway of immunosuppression of CRC tumors.

Considering the recent proof-of-concept published trials demonstrating a potential role of anti-PD1 therapies in a subset of refractory advanced colorectal carcinoma patients, efforts are being done not only to understand the mechanisms in which immune cells can modulate CRC progression but also to find biomarkers to effectively select the patients that could benefit from these new therapeutic approaches. Since NK cell surveillance represents a crucial immune response, investigation of the underlying mechanisms for its functional impairment in CRC patients would open new strategies for effective treatment focusing on NK cell activity.

## Author Contributions

EL conceived and designed the experiments. YR performed all the experiments. YR, MR, EJ, MP, LB, SR, and JM analyzed and interpreted data. EH, FL, AP, and AC contributed with reagents/materials/analysis tools. YR and EL wrote the manuscript. AC and JM corrected the manuscript.

## Conflict of Interest Statement

The authors declare that the research was conducted in the absence of any commercial or financial relationships that could be construed as a potential conflict of interest.

## Supplementary Material

Figure S1 **Gating strategy of NK cell phenotype analysis by FACS**. Expression of NKp30 and its corresponding Isotype control are shown as representative dot plots of thirteen receptors studied.

Figure S2 **Modulation of activation marker CD69 and Natural Cytotoxicity Receptors (NCR) expression on HD-NK cells after overnight IL-2 or IL-15 treatments**.

Figure S3 **CRC-NK and HD-NK relative increase in functionality – lysis (A) or ADCC (B) – due to IL-2 or IL-15 stimulation**. **(C)** CRC-NK and HD-NK relative increase in lysis due to joint stimulation by cetuximab and IL-2 or IL-15.

## Abbreviations

ADCC, antibody-dependent cellular cytotoxicity; CRC, colorectal cancer; CRC-NK, peripheral blood NK cells from colorectal cancer patients; EGFR, epidermal growth factor receptor; HD, healthy donors; HD-NK, peripheral blood NK cells from healthy donors; IFN-γ, interferon-γ; NCR, natural cytotoxicity receptors; PB, peripheral blood; PBMC, peripheral blood mononuclear cells; PFS, progression-free survival; RFS, relapse-free survival; TNF-α, tumor-necrosis factor-α.
