# Supplementary material for: Phenotypic and Functional Dysregulated Blood NK Cells in Colorectal Cancer Patients Can Be Activated by Cetuximab Plus IL-2 or IL-15
Source: Front Immunol. 2016 Oct 10;7:413. doi: 10.3389/fimmu.2016.00413 (PMC5056190; doi:10.3389/fimmu.2016.00413)
Supplement: Supplementary file 1 [file Table_1.PDF]

Supplementary Table 1. List of antibody clones and their sources used.

Antibody	Fluorochrome	Brand	Clone
CD45	PerCP	BD Biosciences	2D1
CD3	FITC	BD Biosciences	UCHT1
CD3	PerCP	BD Biosciences	SK7
CD56	APC	BD Biosciences	NCAM 16.2
CD16	PE	BD Biosciences	3G8
NKG2D (CD314)	PE	BD Biosciences	1D11
DNAM-1 (CD226)	PE	BD Biosciences	DX11
NKp30 (CD337)	PE	BD Biosciences	P30-15
NKp44 (CD336)	PE	BD Biosciences	p44-8.1
NKp46	PE	BD Biosciences	9E2/NKp46
CD161	PE	BD Biosciences	DX12
CD8	PE	BD Biosciences	RPA-T8
CD158a	PE	BD Biosciences	HP-3E4
CD158b	PE	BD Biosciences	CH-L
NKG2A	PE	R & D System	131411
CD85j	PE	BD Biosciences	GHI/75
CD94	PE	BD Biosciences	HP-3D9
CD107a	PE	BD Biosciences	H4A3
IFN-γ	PE	BD Biosciences	4S.B3
NKp30 blocking	----------------------------	R & D System	210845
NKp46 blocking	----------------------------	R & D System	195314
DNAM-1 blocking	----------------------------	R & D System	102511
NKG2D blocking	----------------------------	R & D System	149810
