# Supplementary material for: Phenotypic and Functional Dysregulated Blood NK Cells in Colorectal Cancer Patients Can Be Activated by Cetuximab Plus IL-2 or IL-15
Source: Front Immunol. 2016 Oct 10;7:413. doi: 10.3389/fimmu.2016.00413 (PMC5056190; doi:10.3389/fimmu.2016.00413)
Supplement: Supplementary file 2 [file Table_2.PDF]

		Localization			CEA		Diferentiation		LVI		Stages	
		**Right**	**Left**	**Rectum**	**≤5**	**>5**	**Well**	**Poor/Other**	**No**	**Yes**	**I-II**	**III-IV**
**$CD3^-CD56^+$**	**n**	7	24	12	20	15	37	6	22	21	26	17
	**%**	18.3±9.3	18.9±11.3	19.6±14.3	18.7±11.3	21.1±13.1	18.5±12.1	21.8±8.9	20.7±13.3	17.2±9.6	17.1±13.3	21.8±8.1
	**p**	ns			ns		ns		ns		**0.0469**	
**CD16**	**n**	6	21	7	18	12	27	6	17	16	20	13
	**%**	89.2±6.8	93.4±5.0	96.3±2.2	93.3±4.7	93.2±5.6	92.8±5.7	94.5±3.5	92.7±5.2	93.3±5.7	92.2±5.5	94.5±4.9
	**p**	ns			ns		ns		ns		ns	
**NKG2D**	**n**	9	18	6	20	9	27	6	17	16	20	14
	**%**	89.2±10.5	86.5±22.7	85,2±11.2	87.1±19.7	88.3±16.2	84.8±19.1	96.9±5.4	89.2±12.7	84.7±22.5	85.0±20.8	89.9±12.3
	**p**	ns			ns		**0.0143**		ns		ns	
**NKp30**	**n**	11	18	5	19	10	29	6	15	18	20	15
	**%**	83.9±12.7	88.7±9.7	79.5±18.1	85.8± 12.7	85.1± 13.8	87.7 ± 11.2	78.8± 15.0	84.7± 15.1	86.6 ± 9.9	90.1± 11.3	80.9±11.7
	**p**	ns			ns		ns		ns		**0.0076**	
**CD161**	**n**	8	17	4	15	10	24	5	15	14	18	11
	**%**	63.2±14.8	65.8±29.0	60.1±21.3	62.2±25.7	71.5±25.1	61.2±24.6	79.4±17.2	69.1±23.1	59.8±24.9	65.4±22.1	62.4±28.5
	**p**	ns			ns		ns		ns		ns	
**DNAM-1**	**n**	7	23	3	17	13	29	5	17	15	19	14
	**%**	70.6±29.0	77.3±21.9	73.7±12.8	74.5±22.6	85.6±12.8	73.0±22.9	91.1±8.0	77.7±17.2	72.6±28.3	71.4±24.4	81.1±19.1
	**p**	ns			ns		**0.0411**		ns		ns	
**NKp46**	**n**	7	17	5	16	10	22	6	16	13	18	11
	**%**	77.5±24.1	80.2±17.8	74.5±25.2	77.0±23.0	84.2±12.1	76.4±22.0	83.7±10.1	79.1±21.7	77.9±18.6	81.7±18.2	73.5±22.1
	**p**	ns			ns		ns		ns		ns	
**CD158a/h**	**n**	6	11	5	12	7	20	3	11	12	14	10
	**%**	9.0±9.9	8.6±8.1	8.9±6.1	7.9±8.6	10.6±8.0	8.3±7.4	11.0±10.0	8.8±8.6	8.2±7.0	8.1±7.3	9.2±8.1
	**p**	ns			ns		ns		ns		ns	
**CD94**	**n**	5	14	3	10	9	17	5	12	9	14	8
	**%**	87.3±12.8	86.3±12.7	90.2±9.8	83.9±13.4	93.8±5.2	84.5±12.0	95.8±6.4	88.9±9.5	83.4±14.6	87.5±7.7	86.2±17.7
	**p**	ns			**0.035**		**0.0188**		ns		ns	
**NKp44**	**n**	6	14	4	13	9	17	7	12	11	15	9
	**%**	4.2±3.5	2.0±1.6	1.8±1.6	2.2±2.8	3.0±1.8	2.7±2.4	2.1±2.2	3.3±2.7	1.7±1.8	2.5±2.5	2.6±2.1
	**p**	ns			ns		ns		ns		ns	
**CD85j**	**n**	4	13	3	11	7	20	0	11	9	14	6
	**%**	43.7±31.8	34.0±25.2	60.4±24.5	44.5±28.1	40.8±25.4	39.9±26.8		39.1±23.4	40.9±31.9	37.2±21.4	46.3±38.4
	**p**	ns			ns				ns		ns	
**CD158b**	**n**	7	9	5	15	5	18	3	11	10	14	7
	**%**	30.4±9.9	33.7±16.9	33.7±18.0	31.3±14.7	33.0±13.9	32.9±15.5	34.9±7.8	28.2±14.3	38.0±13.3	29.3±15.0	39.1±11.9
	**p**	ns			ns		ns		ns		ns	
**CD8**	**n**	3	10	4	9	6	17	0	9	8	12	5
	**%**	35.7±22.7	32.9±13.3	55.2±20.1	41.3 ±17.6	43.9 ±14.5	38.6±18.2		49.3±15.6	26.6±12.9	41.8±19.6	31.2±12.9
	**p**	ns			ns				**0.0054**		ns	
**NKG2A**	**n**	6	19	5	19	8	24	5	16	14	19	11
	**%**	43.3±16.6	36.0±16.5	25.5±14.8	33.6±14.1	46.5±19.3	36.3±11.2	39.1±32.0	42.2±16.0	28.3±14.5	38.1±14.1	31.5±20.4
	**p**	ns			ns		ns		**0.0196**		ns	

Supplementary table 2. Association of clincal features displayed by CRC patients and NK cell receptor expression.
Abbreviations: CEA= carcino-embryonic antigen; LVI= lymphovascular invasion.
