# Supplementary material for: Phenotypic and Functional Dysregulated Blood NK Cells in Colorectal Cancer Patients Can Be Activated by Cetuximab Plus IL-2 or IL-15
Source: Front Immunol. 2016 Oct 10;7:413. doi: 10.3389/fimmu.2016.00413 (PMC5056190; doi:10.3389/fimmu.2016.00413)
Supplement: Supplementary file 3 [file Table_3.PDF]

Supplementary Table 3. HD-NK cell receptor modulation by IL-2 or IL-15 overnight treatment (n=3).

Receptor	Initial		IL-2		IL-15	
	**%**	**MFI**	**%**	**MFI**	**%**	**MFI**
CD16	89,5 ± 3,4	1394 ± 640	91,3 ± 4,1	2341,0 ± 779,0	89,4 ± 3,8	2097,0 ± 545,0
NKG2D	80,2 ± 5,2	45,9 ± 9,6	93,0 ± 2,9	89,2 ± 18,9	97,0 ± 0,4	117,4 ± 19,4
NKp30	74,8 ± 16,4	39,6 ± 3,1	80,3 ± 6,4	62,9 ± 8,1	90,1 ± 3,8	84,3 ± 17,2
NKp44	19,3 ± 9,5	30,5 ± 6,7	33,2 ± 13,5	56,4 ± 16,9	46,0 ± 12,8	62,3 ± 17,1
NKp46	91,8 ± 5,4	98,2 ± 22,5	89,2 ± 6,3	81,6 ± 10,9	82,7 ± 9,1	77,2 ± 17,9
DNAM-1	82,3 ± 5,6	41,1 ± 7,3	85,4 ± 3,8	50,0 ± 3,2	84,7 ± 6,3	46,0 ± 5,3
CD94	85,1 ± 4,7	198,0 ± 46,7	95,7 ± 1,5	280,0 ± 61,0	97,3 ± 1,0	323,0 ± 59,0
CD85j	30,6 ± 14,6	22,0 ± 3,0	29,3 ± 16,0	20,6 ± 2,4	33,3 ± 16,0	21,5 ± 0,5
CD158a	8,1 ± 1,9	19,7 ± 1,6	12,8 ± 3,0	22,1 ± 5,1	14,0 ± 6,7	30,5 ± 4,8
NKG2A	17,2 ± 1,8	22,8 ± 1,9	15,9 ± 0,6	24,5 ± 2,9	19,5 ± 4,5	29,5 ± 3,0
CD158b	26,5 ± 8,3	139,0 ± 20,0	28,1 ± 7,4	159,0 ± 26,0	28,1 ± 7,3	181,0 ± 42,0
CD161	59,5 ± 3,6	33,6 ± 2,6	64,9 ± 7,3	40,0 ± 5,7	67,4 ± 3,2	38,3 ± 5,7
CD69	0,75 ± 0,14	10,8 ± 0,4	16,0 ± 3,6	12,8 ± 0,6	26,0 ± 2,3	13,6 ± 0,3
