# Supplementary figures and images for: Phenotypic and Functional Dysregulated Blood NK Cells in Colorectal Cancer Patients Can Be Activated by Cetuximab Plus IL-2 or IL-15
Source: Front Immunol. 2016 Oct 10;7:413. doi: 10.3389/fimmu.2016.00413 (PMC5056190; doi:10.3389/fimmu.2016.00413)

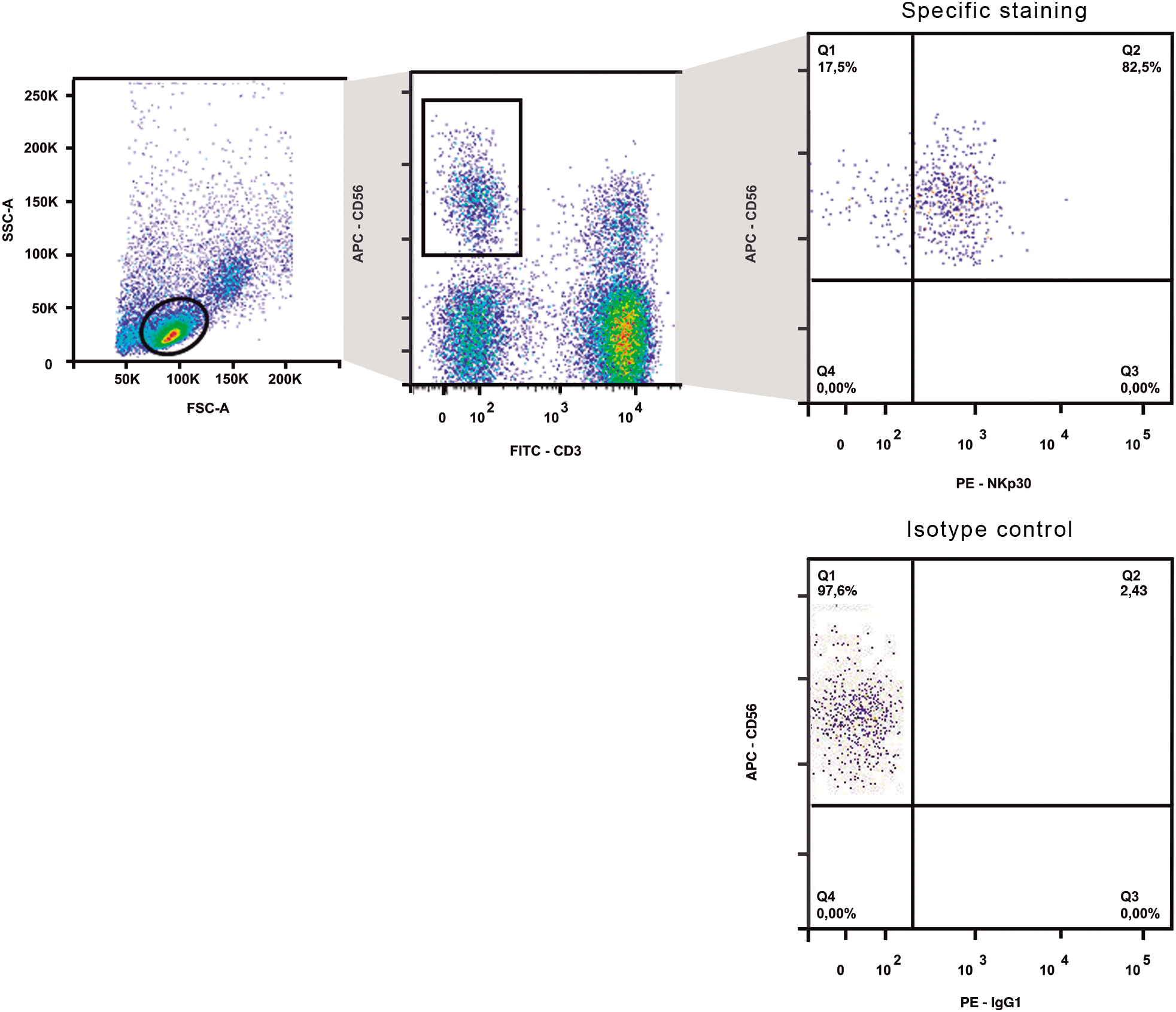

Supplement: Figure S1 — Gating strategy of NK cell phenotype analysis by FACS. Expression of NKp30 and its corresponding Isotype control are shown as representative dot plots of thirteen receptors studied. [file Image_1.TIF]

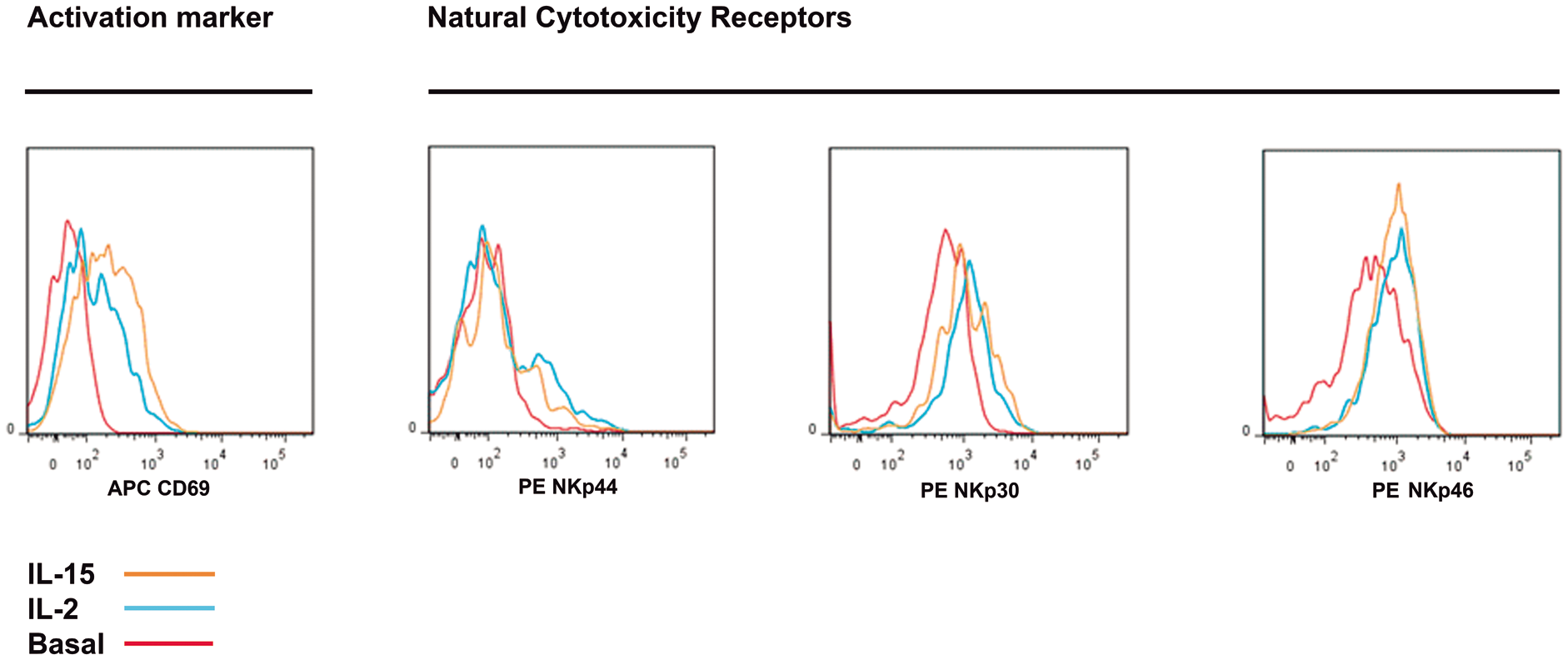

Supplement: Figure S2 — Modulation of activation marker CD69 and Natural Cytotoxicity Receptors (NCR) expression on HD-NK cells after overnight IL-2 or IL-15 treatments. [file Image_2.TIF]

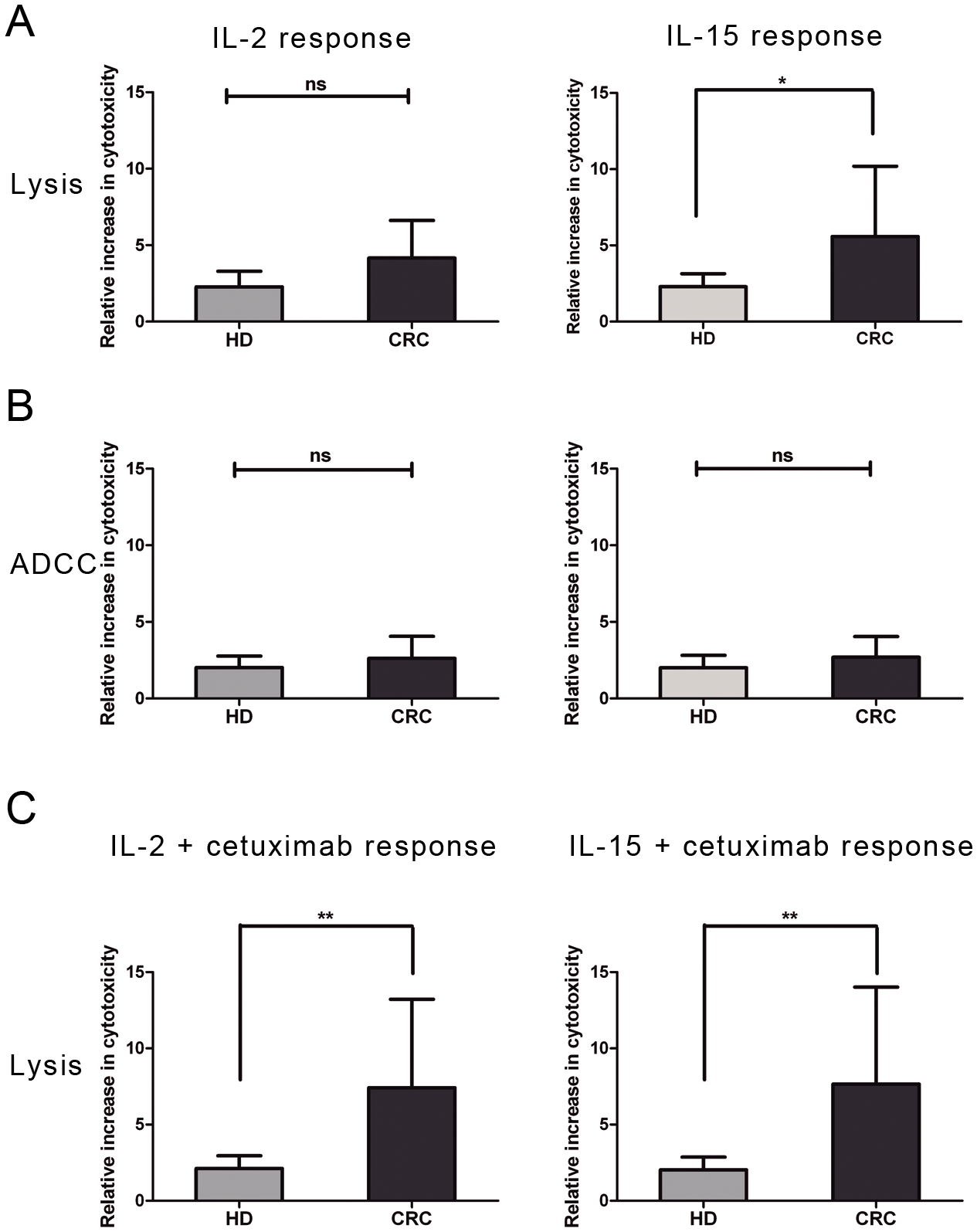

Supplement: Figure S3 — CRC-NK and HD-NK relative increase in functionality – lysis (A) or ADCC (B) – due to IL-2 or IL-15 stimulation. (C) CRC-NK and HD-NK relative increase in lysis due to joint stimulation by cetuximab and IL-2 or IL-15. [file Image_3.TIF]
